# Xmas‐NT: A Novel Two‐Color Fluorescence Micro Neutralization Test for the Detection of Neutralizing Antibodies Against Monkeypox Virus

**DOI:** 10.1002/jmv.70246

**Published:** 2025-02-20

**Authors:** Clemens Bodenstein, Daniel Bourquain, Andreas Nitsche, Marica Grossegesse

**Affiliations:** ^1^ Highly Pathogenic Viruses (ZBS 1), Centre for Biological Threats and Special Pathogens WHO Collaboration Center for Emerging Threats and Special Pathogens, Robert Koch Institute Berlin Germany

**Keywords:** micro neutralization assay, monkeypox virus, neutralizing antibodies

## Abstract

Since the global outbreak of Mpox in 2022, interest in seroepidemiological and vaccine studies has grown rapidly. One aspect of interest in these studies is the detection of neutralizing antibodies to the causative agent, monkeypox virus (MPXV). Determination of neutralizing antibodies using a (micro)‐neutralization test (NT) is the gold standard method, which is time consuming and labor intensive. Here, we present the comparison of different methods to facilitate MPXV NT analysis resulting in the establishment of an optimized MPXV NT protocol. The optimized protocol is based on a recombinant eGFP‐expressing MPXV and a recombinant mCherry‐expressing VeroE6 cell line. Using the so‐called Xmas‐NT, the incubation time can be reduced by 60%. In addition, compared to the conventional NT, the documentation time of the Xmas‐NT is significantly reduced by 80%, making it suitable for serological high throughput screening studies.

## 1 Introduction

Monkeypox virus (MPXV) belongs to the genus of *Orthopoxvirus* (OPXV) and is the causative agent of Mpox [1]. Mpox is characterized by headache, muscle pain, fever and typical skin lesions [2]. Originally a zoonotic disease, Mpox was endemic in Central and Western Africa. However, since 2022, more human‐to‐human transmissions through close skin or mucosal contact have occurred [3]. By 2022/2023 Mpox has spread to more than 100 countries worldwide and since early 2023 a major Mpox outbreak is taking place in the DRC with more than 43 000 suspected Mpox cases and more than 1100 deaths as of December 1, 2024 [4].

Considering Mpox as an emerging disease, this raises questions about the potential immunological protection of the population. To answer these questions, seroepidemiological studies must be carried out. Neutralizing antibodies are of special interest, because they may confer protection. However, the challenge is the detection of these neutralizing antibodies, which is laborious and time‐intensive. In a recent publication we described the establishment of a micro neutralization test (NT) for MPXV [5]. Briefly, patient serum is incubated with a specific amount of MPXV to allow antibodies to bind to the virus. The mixture is then applied to a cell monolayer and incubated for 7 days. If neutralizing antibodies are present in the serum, they will prevent the virus from infecting the cells. Conversely, if no neutralizing antibodies are present in the serum, the virus can infect the cells and cause visible damage to the cell monolayer (CPE, cytopathic effect), which is then manually/visually analyzed well by well under a light microscope. The drawback of this test is the low throughput, the long incubation time (7 days) and the labor‐intensive visual analysis (about 5 min per sample). We therefore aimed to optimize the test in a way that it could be applied in seroepidemiological and vaccine studies to analyze the presence of neutralizing antibodies in larger cohorts in a manageable amount of time.

We started with three different optimization approaches aimed at reducing incubation time and simplifying analysis. For the first approach, we generated a green fluorescent protein (GFP)‐expressing MPXV (GFP‐MPXV, GenBank PQ271634), whose proliferation in cell culture should be easier and faster detectable using a fluorescence microscope compared to the CPE readout using a light microscope. For the second approach, we generated a cell line constitutively expressing a red fluorescent protein (mCherry) to increase the signal (CPE) to background (cell) contrast. Thirdly, we combined the two approaches by using the GFP‐MPXV on mCherry‐VeroE6 cells. All three setups were then compared with our original published protocol to determine the earliest possible time point for analysis. The optimized protocol using both GFP‐MPXV and mCherry‐VeroE6 was then further evaluated and compared to the original protocol in biological and technical triplicates, demonstrating a reduction of incubation time down to 3 days. Because of the colors (green‐red), we call the optimized protocol “Xmas‐NT”.

## 2 Materials and Methods

### 2.1 Cell Culture

VeroE6 cells (#85020206, ECACC) were cultured in growth medium (DMEM with 5% FBS and 2 mM l‐glutamine). mCherry‐VeroE6 cells were cultured in selection medium (DMEM with 5% FBS, 2 mM l‐glutamine and 7 µg/mL puromycin). All cells were kept at 37°C, 5% CO_2_. For neutralization tests, cells were seeded in 96‐well plates (Nunclon Delta surface) with a density of 1.5 × 10^5^ cells/mL at 100 µL/well (total cell count using automated cell counter Countess 3).

### 2.2 Samples

Serum samples from PCR‐confirmed MPXV‐infected individuals were collected in the course of the routine diagnostics in the Consultant Laboratory for Poxviruses at the Robert Koch Institute. Ethical clearance was obtained from the Berliner Ärztekammer (BÄK Eth‐44/22).

### 2.3 Generation of Recombinant MPXV

Recombinant GFP‐expressing MPXV (GFP‐MPXV) were generated through homologous recombination and subsequent plaque purification as previously described by Byrd et al. [6] The in‐house isolate MPXV clade IIb strain (wtMPXV, MPXV/Germany/2022/RKI01) was chosen as a starting material for recombination [5]. eGFP under control of the strong viral early/late promoter combined with a 99 nt spacer was inserted in place of the viral thymidine kinase (TK) ORF [7, 8]. To achieve the homologous recombination, VeroE6 cells were simultaneously transfected with a vector plasmid (pEX‐A258, containing the TK‐flanked viral early/late promoter‐eGFP sequence) and infected with the wtMPXV. Three days postinfection (dpi) cells were harvested. GFP‐MPXV was isolated throughout 5 rounds of plaque purification. The purified GFP‐MPXV was propagated on VeroE6 cells, sequenced and stocks for neutralization assay (target titer of 1000 TCID50/mL) were prepared and stored at −70°C.

### 2.4 Generation of a Recombinant VeroE6 Cell Line

A recombinant VeroE6 cell line (mCherry‐VeroE6) was generated using a lentiviral vector (GeneCopoeia, LP441‐025‐GVO‐GC), that contained mCherry controlled by a CMV promoter and a puromycin resistance gene as a selection marker. Cells were transduced with DMRIE‐C following the standard protocol of the manufacturer. Three days posttransduction regular growth medium was exchanged for selection medium. Plates were checked every 3 days for recombinant colonies and selection medium was exchanged for fresh selection medium. Recombinant colonies were isolated with cloning cylinders and seeded in new plates until all cells showed mCherry signal.

### 2.5 Conventional NT

NTs were done with wtMPXV (MPXV clade IIb, MPXV/Germany/2022/RKI01) and wtVeroE6 as described in a previous paper with a minor adaptation [5]. Cells were cultivated in DMEM with 5% FBS rather than 10% FBS. Briefly, patient sera were diluted in DMEM to six twofold dilution steps, resulting in dilutions of 1:10–1:320. Equal amounts of wtMPXV stock (target titer of 1000 TCID_50_/mL) were added to each dilution step and mixed, resulting in final dilutions from 1:20 to 1:640. After 1 h of incubation, each virus‐neutralizing antibody mix was added to 8 wells of a 96‐well plate with seeded wtVeroE6 cells. NTs with wtMPXV were examined for CPE after 7 days with a widefield microscope (EVOS Core XL). CPE analyzation was based on subjective interpretation of trained lab personnel. A well was considered positive upon the detection of a single, unambiguous plaque. Titers were calculated following the formula: Titer (1:x) = 10 × 2^(*n*/8+0.5)^ where *n* = number of negative wells. When no CPE could be detected, the titer was marked as threshold titer (1: ≤ 14). A parallel titration for titer confirmation of the virus stock (back‐titration) was done with every NT: Virus stock was diluted 1:1 in DMEM, incubated for 1 h and further diluted in 10‐fold steps. Each dilution was added to 8 wells of a 96‐well plate with VeroE6 cells and CPE was analyzed after 7 days.

### 2.6 Micro‐NT Adaptations

Different adaptions were tested to optimize the NT. Either wtMPXV was replaced by GFP‐MPXV and/or wtVeroE6 cells were replaced by mCherry‐VeroE6. Documentation was done at 3, 5, and 7 dpi. Plate‐wide pictures were taken with the ×1.6 objective of a fluorescence microscope (Leica Mica). Fluorescence‐images were photographed with the following setting: LED 470 with 21.1% intensity, LED 555 with 97.9% intensity, each image was exposed for 100 ms and pictures taken with a gain of 5.0. Subjective CPE interpretation was based on fluorescent plaques. The Xmas‐NT is the optimized NT using GFP‐MPXV and mCherry‐VeroE6 cells and is documented 3 dpi.

## 3 Results

**Figure 1:**
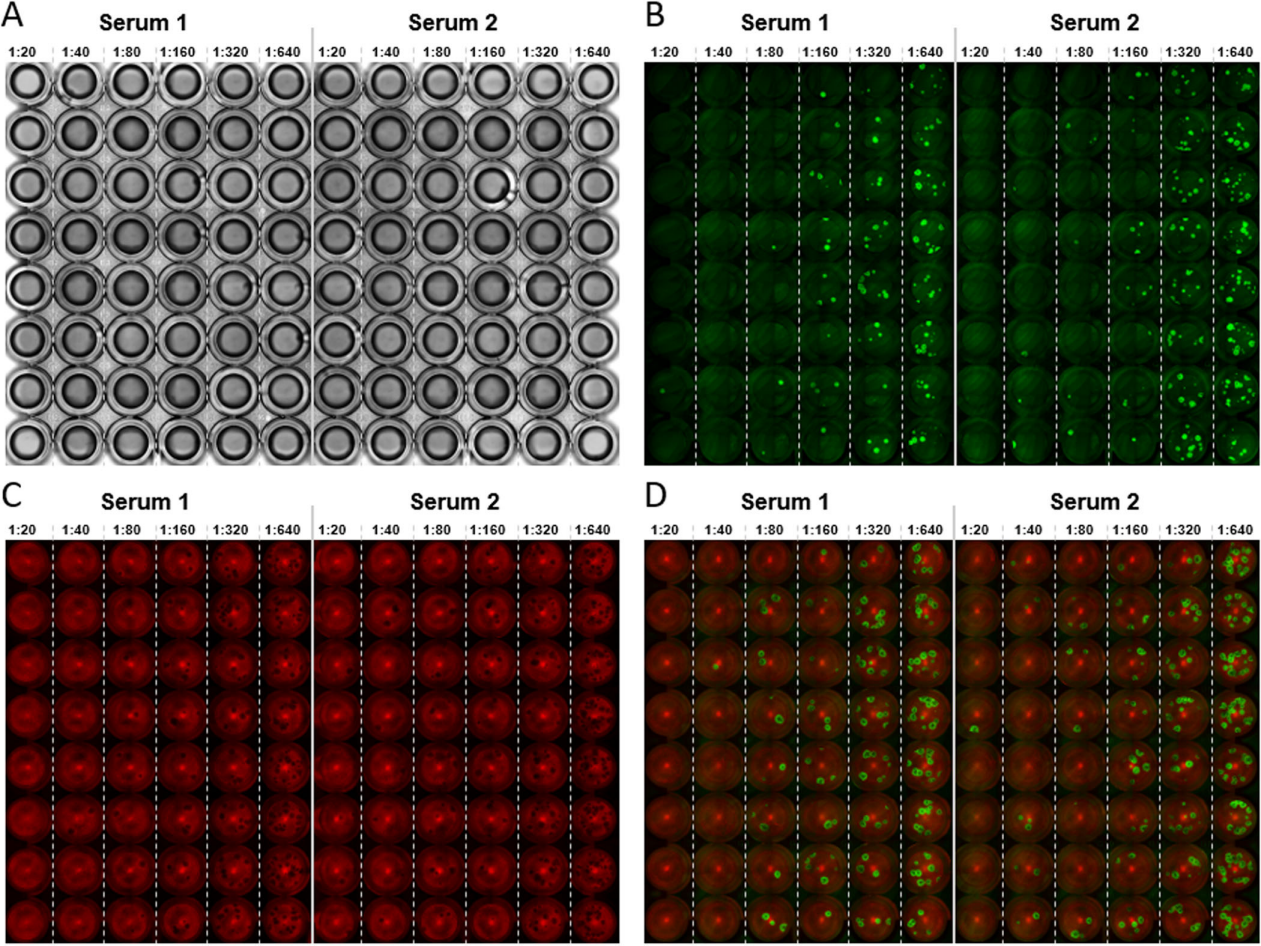
Exemplary images of different micro‐NT setups compared in this study. (A) Original setup used previously (wtMPXV and wtVeroE6 cells). (B) Setup with GFP‐MPXV and wtVeroE6. Infected cells show eGFP signal. (C) Setup with wtMPXV and mCherry‐VeroE6. (D) Xmas‐NT setup with GFP‐MPXV and mCherry‐VeroE6. Infected cells show eGFP signal, cellular background with mCherry signal. The readout of all micro‐NT setups is plaque‐based CPE detection either in transmitted (A), eGFP (B), mCherry channel (C), or merged eGFP/mCherry channel (D). Pictures were taken 3 dpi. Dilution steps are indicated (1:20 to 1:640).

**Figure 2:**
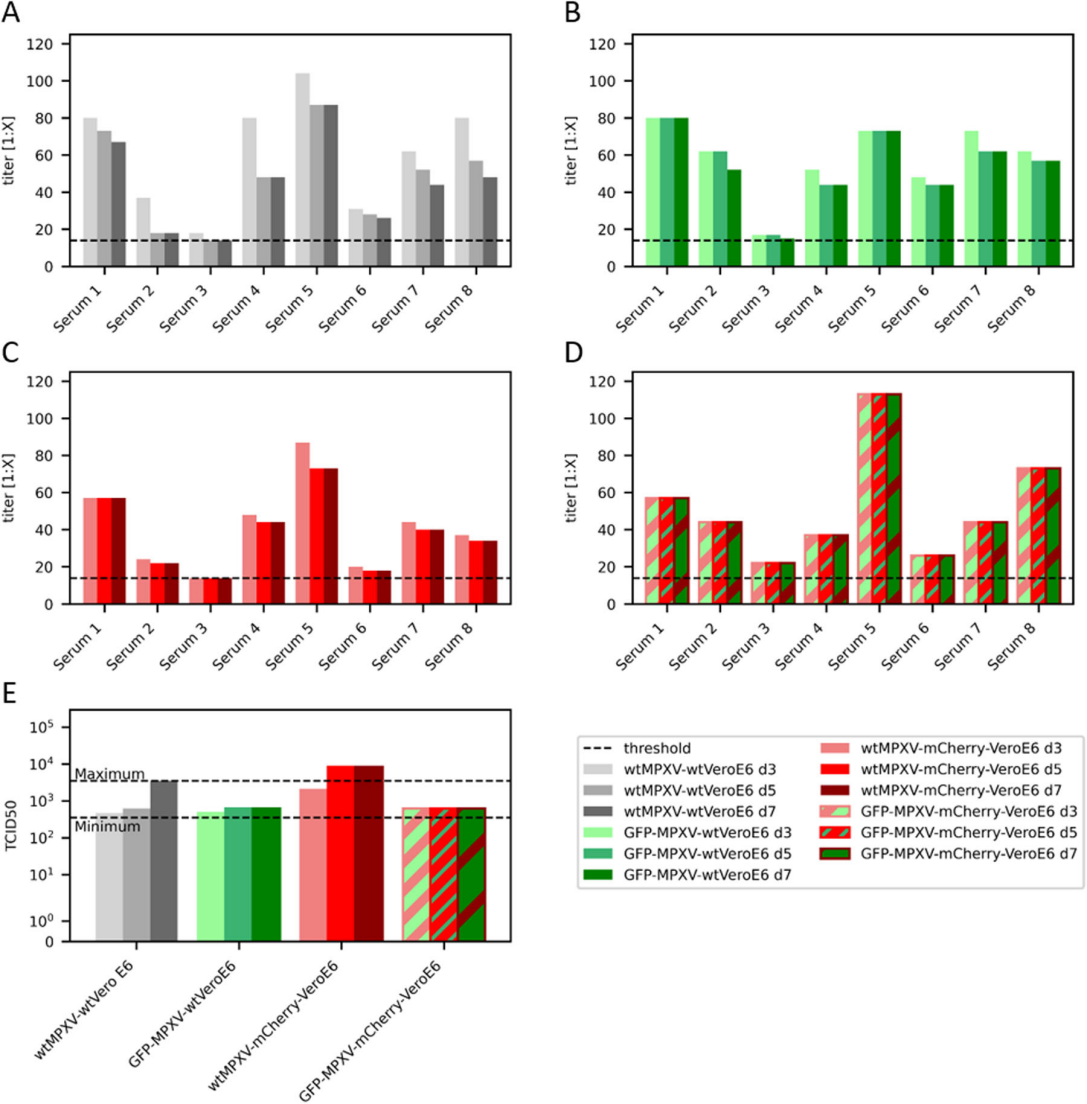
Comparison of different micro‐NT protocols for the detection of neutralizing antibodies against MPXV. Eight sera of PCR‐confirmed MPXV‐positive patients were analyzed in (A) conventional NT with wtMPXV and wtVeroE6, (B) GFP‐MPXV and wtVeroE6, (C) wtMPXV and mCherry‐VeroE6 or (D) GFP‐MPXV and mCherry‐VeroE6 (Xmas‐NT). Briefly, 1000 TCID50/mL of either wtMPXV or GFP‐MPXV were added to diluted sera and incubated for 1 h before infection of either wtVeroE6 or mCherry‐VeroE6 cells. The CPE was documented on different days (3, 5, and 7 dpi) using (A) light or (B–D) fluorescence microscopy to determine the earliest day for accurate and consistent readout (7 dpi represents the standard incubation time of the conventional NT). The detection threshold of the test is indicated with a dashed line (A–D, titer 1:14). (E) shows the results of the back titrations (TCID50 titers) of the virus stocks performed in parallel with the NTs. Dashed lines in (E) represent the target titer range of the back titration, which was previously determined for wtMPXV using 20 independent replicates.

For the initial proof of concept, eight sera with previously confirmed neutralizing capacity against MPXV were analyzed in the micro‐NTs with the four different conditions: wtMPXV with wtVeroE6 (conventional micro‐NT protocol, Figure 1A), GFP‐MPXV with wtVeroE6 cells (Figure 1B), wtMPXV with mCherry‐VeroE6 cells (Figure 1C) and GFP‐MPXV with mCherry‐VeroE6 (Figure 1D). Figure 2 shows the neutralization titers determined for each of the 8 sera documented on 3, 5, and 7 dpi with the different setups (Figure 2A–D). The conventional NT was documented manually/visually, while the others were documented by taking plate‐wide assembled pictures in the GFP‐ and mCherry channel with a fluorescence microscope (Leica Mica) (Figure 1A–D). In the conventional NT, the CPE is documented 7 dpi. In the following, it should be investigated whether an earlier readout is possible. In the conventional NT (Figure 2A), all sera showed a higher neutralizing titer after 3 dpi than at 5 dpi and titers decreased further on 7 dpi for 4 out of 8 sera. This titer reduction demonstrates that the CPE of wtMPXV is not well detectable in wtVeroE6 at early time points (3 dpi) resulting in calculated system‐inherent higher neutralizing titers. In contrast, 4 sera analyzed in the NT with GFP‐MPXV and wtVeroE6 had lower titers at 5 dpi in comparison to 3 dpi and 2 sera decreased in titer from 5 to 7 dpi. This indicates that plaques generated by GFP‐MPXV have a better visibility and can be detected earlier (Figure 1B,D). In the NT with wtMPXV on mCherry‐VeroE6 cells the determined titer changed for 6 out of 8 sera after 5 dpi. However, no changes were observed between 5 and 7 dpi, indicating that the mCherry signal in the cells facilitates plaque recognition and a readout can be done at 5 dpi. Finally, the Xmas‐NT showed consistent results for all of the 8 sera tested between the readouts at 3, 5, and 7 dpi, suggesting that readout is already possible at 3 dpi. A similar trend can be seen in the back titration of the virus stocks used in the NT. WtMPXV on wtVeroE6 showed a clear time‐dependence of the results and the highest titer differences between incubation for 3 and 7 days. For both GFP‐MPXV on wtVeroE6 cells and wtMPXV on mCherry‐VeroE6 cells titers did not change at 5 dpi. Viral titers of GFP‐MPXV determined on mCherry‐VeroE6 cells did not change after 3 dpi.

In addition to the reduced incubation time, the fluorescence‐based NTs also showed a reduced CPE documentation time. Generating a picture of an entire plate took the microscope around 2 min and negative wells without CPE were easily recognizable in seconds.

**Figure 3:**
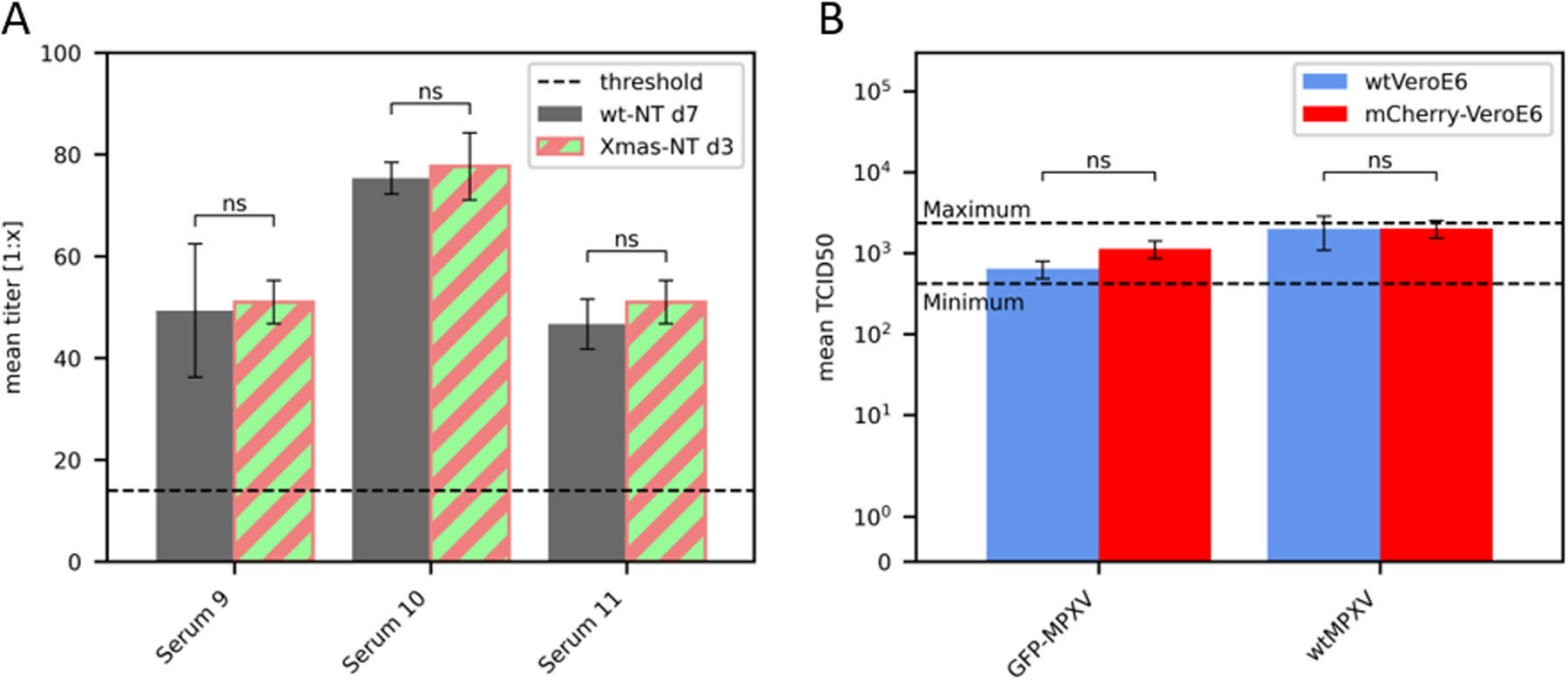
Replicate comparative analysis of the conventional and Xmas‐NT protocol. (A) Comparison of mean titers (*n* = 3) determined following the conventional (wt) NT protocol versus the novel Xmas‐NT. The detection limit of the test (threshold) is indicated with a dashed line (titer 1:14). (B) Comparison of mean titers (*n* = 3) of GFP‐MPXV and wtMPXV virus stocks determined by titration on wtVeroE6 cells and mCherry‐VeroE6 cells. Dashed lines indicate the target titer range. Statistics revealed no significant differences (two‐tailed *t*‐test, *p* > 0.05).

One aspect to note when comparing the approaches was the variability in titers for the same sera documented in the different NTs (e.g., Serum 6 had a titer of 1:18 in the conventional NT and a titer of 1:44 in the Xmas‐NT). From our routinely performed conventional MPXV micro‐NT we know that variations of up to ±3 wells are to be expected. To analyze if titers determined by our new Xmas‐NT approach are comparable to the conventional NT, 3 sera were tested 3 times each in the conventional NT (documentation after 7 dpi) and in the Xmas‐NT (documentation on 3 dpi). As shown in Figure 3A, the mean titers of the sera tested between the two approaches were well comparable and differences were insignificant (two‐tailed *t*‐test, *p* > 0.05). Titrations of the virus stocks were also performed in triplicates on either VeroE6 or mCherry‐VeroE6 cells to confirm that the titers are within the expected range (Figure 3B). The target NT stock titer is 1.12 × 10^3^ TCID_50_/mL with an accepted range from 4.22 × 10^2^ TCID_50_/mL to 2.37 × 10^3^ TCID_50_/mL corresponding to ±3 positive wells. The mean titers of both stocks were within the accepted range and also differences for each stock were again insignificant between titration on wtVeroE6 cells and mCherry‐VeroE6 cells (two‐tailed *t*‐test, *p* > 0.05).

**Figure 4:**
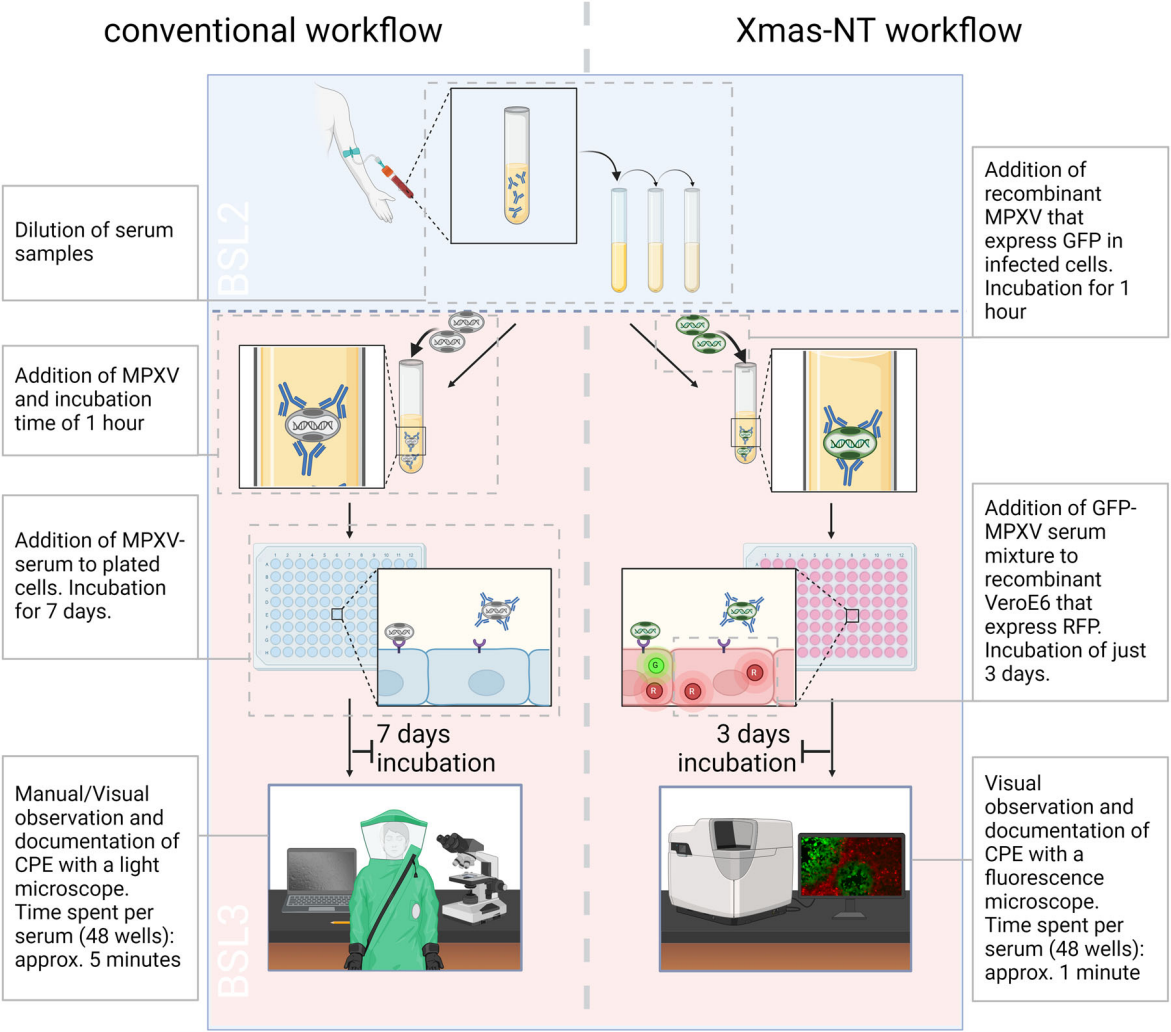
Schematic workflow of the conventional NT compared to the optimized Xmas‐NT. Using the Xmas‐NT workflow, the incubation time can be reduced from 7 to 3 days. Furthermore, the optimized workflow reduces the time required for documentation to approximately 1 minute per sample. BioRender.com/y49r577.

Finally, after showing the comparability of the results of the Xmas‐NT and the conventional NT, both workflows are summarized and compared in Figure 4.

## 4 Discussion

The prevalence of neutralizing antibodies is considered to be a good predictor of immunological protection against OPXV infections [9, 10]. For this purpose, the neutralizing capacity of serum samples is determined in the laboratory by NT. While the general principle of NTs is similar—mixing virus with serum and applying it on cells—the way of analysis can be different. For poxviruses, the classic way of analysis is to stain the cells with a dye and then inspect for CPE. This method (plaque reduction neutralization assay) is laborious and requires prior inactivation and fixation of the infected cell monolayer. Therefore, it is not suitable for larger screening studies and may, hence, be replaced by other analysis methods, e.g. testing for viral mRNA by qPCR instead of looking at visible CPE [11]. While detection of the viral RNA could increase test sensitivity, analyzing each well in a separate PCR assay is also time and material consuming. Currently, staining infected cells with antibodies is a popular analysis method for NT [9, 12]. It is also possible to analyze the neutralization capacity using a reporter‐gene, such as beta‐galactosidase or GFP, which may be detected via microplate readers or flow cytometry [13, 14, 15].

In this manuscript, we present an optimized MPXV micro‐NT called Xmas‐NT. The Xmas‐NT utilizes a combination of a GFP‐expressing virus and mCherry‐expressing cells, significantly simplifying the readout process by eliminating the need for antibody staining or additional detection steps for infected cells. By merging the distinct fluorescent signals, the contrast of infected cells forming plaques within the cell monolayer is enhanced, enabling faster and more straightforward detection. This reduces the incubation time required for unambiguous plaque identification. In conventional NTs where the readout relies on visible CPE, a significantly longer incubation time is needed for clear plaque identification. Xmas‐NT can be easily adapted for large‐scale analysis of MPXV neutralizing antibodies, e.g. by reducing the number of dilutions and replicates, making it suitable for high‐throughput screening. In the future, we plan to apply the Xmas‐NT to a larger seroepidemiological MPXV study to further validate its high‐throughput capabilities. In addition, Xmas‐NT can be performed with other MPXV isolates and variants (GFP variants of other clades are currently in preparation). Finally, the generated mCherry‐VeroE6 cells could be used in combination with viruses other than MPXV that show a CPE to facilitate the detection of neutralizing antibodies.

## Author Contributions

Conceptualization: Marica Grossegesse and Daniel Bourquain. Resources and data curation: Clemens Bodenstein, Marica Grossegesse, and Andreas Nitsche. writing—original draft preparation: Marica Grossegesse and Clemens Bodenstein. Visualization: Clemens Bodenstein and Marica Grossegesse. Supervision: Andreas Nitsche. All authors reviewed the article and have agreed to the published version of the manuscript.

## Conflicts of Interest

The authors declare no conflicts of interest.

## Data Availability

The data that support the findings of this study are available from the corresponding author upon reasonable request.
